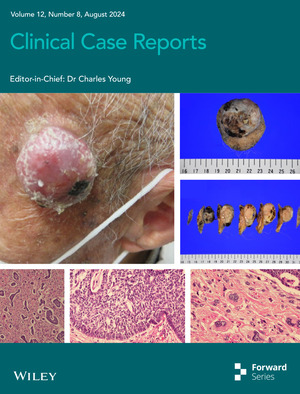# Cover Image

**DOI:** 10.1002/ccr3.9433

**Published:** 2024-09-12

**Authors:** Mariko Suzuki‐Ueno, Yoshiaki Fujikawa, Dai Hamaoka, Kaoru Umemura, Takamasa Ohnishi

## Abstract

The cover image is based on the Article *A collision tumor of basal cell carcinoma and atypical fibroxanthoma: A case report* by Mariko Suzuki‐Ueno et al., https://doi.org/10.1002/ccr3.9250